# Genetic Variants in Group-Specific Component (GC) Gene Are Associated with Breast Cancer Risk among Chinese Women

**DOI:** 10.1155/2019/3295781

**Published:** 2019-11-15

**Authors:** Fuxing Chen, Zheng Zhu, Fränzel J. B. van Duijnhoven, Meihua Dong, Yun Qian, Hao Yu, Jie Yang, Lan Cui, Renqiang Han, Jian Su, Wencong Du, Jinyi Zhou, Ming Wu

**Affiliations:** ^1^Department of Epidemiology and Biostatistics, School of Public Health, Nanjing Medical University, Nanjing 211166, China; ^2^Department of Chronic Disease Control, Jiangsu Provincial Center for Disease Control and Prevention, Nanjing 210009, China; ^3^Division of Human Nutrition and Health, Wageningen University & Research, Wageningen, Netherlands; ^4^Department of Chronic Disease Control, Wuxi Center for Disease Control and Prevention, Wuxi 214023, China

## Abstract

The group-specific component (GC) gene, one of the vitamin D pathway genes, seems to play an important role in cancer development. A population-based breast cancer study including 818 cases and 935 controls in a Chinese population was carried out to evaluate the potential associations of four polymorphisms (rs16847024, rs17467825, rs2298850, and rs3755967) in the GC gene with risk of breast cancer. We detected three SNPs with statistically significant effects on breast cancer development after adjusting for age, menopausal status, body mass index (BMI), family history of breast cancer, income, waist circumference, and education (rs17467825: adjusted OR = 0.80, 95% CI = 0.65–0.99; rs2298850: adjusted OR = 0.80, 95% CI = 0.65–0.98; rs3755967: adjusted OR = 0.80, 95% CI = 0.65–0.98). Stratified analysis found that when an individual had a waist circumference <80 cm, rs17467825, rs2298850, and rs3755967 could markedly reduce the risk of breast cancer. Significant interactions between polymorphisms of rs2298850 and rs3755967 and waist circumference were also observed for breast cancer risk. Combined analysis revealed a significant association among the allele numbers of protective effects with decreased breast cancer risk (*P*_trend_=0.043). These results indicated that, in the GC gene, genetic mutations might be related to breast cancer susceptibility in Chinese women.

## 1. Introduction

Breast cancer is the common therioma in women around the world, with an estimation of 2.1 million new cases and 626,679 deaths [1]. Similar with other countries, breast cancer has become the most frequent carcinoma among the Chinese women. In China, patients with breast cancer make up 11.2% of newly diagnostic cases and 9.2% of breast cancer-related deaths worldwide [2]. As in most other diseases, besides behavioral factors and environmental exposures, genetic variations also make an essential contribution to breast cancer occurrence as well as risk prediction.

A growing number of epidemiological researches have reported that vitamin D could lower the breast cancer risk in humans [3–5]. Vitamin D, as a prohormone, can be changed in the liver into 25(OH)D which can be further hydroxylated to 1,25(OH)_2_D by 1*α*-hydroxylase in the kidney [6]. Vitamin D binding protein (DBP), a pivotal protein, is encoded by the group-specific component gene (GC gene), which is 42.5 kb long and located on chromosome 4 [7]. The functions of DBP are to bind 25(OH)D to prolong its half-life in circulation, as well as transport 25(OH)D to different sites to facilitate vitamin D physiological roles, such as cell proliferation, apoptosis, and differentiation [8]. DBP is highly polymorphic serum protein, characterized by three common alleles (GC1F, GC1S, and GC2) and more than 120 identified rare variants [9]. GC1F, GC1S, and GC2 from rs7041 and rs4588 combination differ in binding affinities with 25(OH)D and therefore result in alteration of serum 25(OH)D concentration [10], which may correlate with risk of diseases, like diabetes, melanoma, and breast cancer [11–13].

Previous studies on the GC gene polymorphism mainly focused on rs7041 and rs4588 and have demonstrated their associations with the risk of different cancers [14, 15]. Elkum [16] et al. revealed that homozygous variants of rs17467825, rs2298850, and rs3755967 were associated with lower levels of serum 25(OH)D among Arabs and South Asians, whereas decreased serum 25(OH)D levels have been shown to be tightly associated with increased risk of several malignant tumors [17, 18]. In addition, Pibiri et al. found that rs16847024 could affect individual susceptibility to colorectal cancer [19]. Currently, the associations between these genetic variants (rs16847024, rs17467825, rs2298850, and rs3755967) and breast cancer risk remain absent due to investigation; based on the previous findings, we reasonably hypothesized that they might contribute to breast carcinogenesis and thus explore the mutual relations among the Chinese women in this study.

## 2. Materials and Methods

### 2.1. Statement of Ethics

The Ethics Committee of Jiangsu Provincial Center for Disease Control and Prevention approved this work. All study subjects were voluntary and have completed written informed consent.

### 2.2. Study Participants

The subjects including 1,410 cases and 1,072 cancer-free controls were recruited between 2013 and 2014 in the largest Wuxi Maternal and Child Health Hospital. All breast cancer cases were newly diagnosed. The exclusion criteria were as follows: (1) a metastatic carcinoma from other sites, (2) a recent history of severe acute diseases, and (3) failed to complete questionnaires. As a result, 818 cases were included. Controls resided in the same areas and were frequency matched with cases by age (within 5 years old); a total of 935 controls were finally included in the present analysis. Standard questionnaires were used to interview each participant to acquire information about demographic data, family history of breast cancer, reproductive and menstrual history, and relevant factors. Several anthropometric measurements, such as height, weight, and waist circumference, were collected during interview stages. Each participant provided approximately 5 ml venous blood sample for subsequent laboratory measurements.

### 2.3. Genotyping

Total DNA was isolated from the blood sample with the use of QIAamp DNA Blood Mini Kit (QIAGEN, Germany) and stored in a −80°C refrigerator. The Sequenom MassARRAY Platform was applied to perform genotyping without knowing the status of subjects. Around 5% of samples were randomly selected for quality control and regenotyped by this platform, and the two results were consistent. Haploview 4.2 was used to examine the linkage disequilibrium of four SNPs. The detailed information of four SNPs in the GC gene is displayed in [Supplementary-material supplementary-material-1].

### 2.4. Statistical Analysis

Differences between cases and controls in the demographic characteristics and selected variables were tested using student's *t*-test (for continuous variables) or Welch's *t*-test (for the situations of unequalled variances) and *χ*2 test (for categorical variables). Logistic regression was applied to evaluate the associations between four SNPs in GC gene and risk of breast cancer. Odds ratios (ORs) and 95% confidence intervals (CIs) were presented with adjusting for age, menopausal status, body mass index (BMI), family history of breast cancer, income, waist circumference, and education. The effect heterogeneity between corresponding subgroups was measured by using the χ2-based Q-test. Three additive interaction measures [20] were calculated by the epiR packages: (1) RERI: relative excess risk due to interaction; (2) AP: attributable proportion due to interaction; and (3) S: synergy index. 95% CIs of RERI and AP across 0 as well as S across 1 indicate no additive interaction effect. The multiplicative interaction of gene environment was also analyzed using logistic regression by the function as described previously [21]:

(1) $$\begin{array}{c} y=b_{0}+b_{1}\times g+b_{2}\times e+b_{3}\times \left(g\times e\right), \end{array}$$

in which *y* is the case or control logit status, *g* represents the SNP, *e* indicates the environment factor: waist circumference, *b*_0_ is the constant, *b*_1_ and *b*_2_ are main effects for *g* and *e*, respectively, and *b*_3_ is the multiplicative interaction term. *P* < 0.05 was as a significant level with two-sided among all statistical tests. Combined analysis was applicable to evaluate the cumulative effect of protective alleles from significant SNPs (rs17467825, rs2298850, and rs3755967). Statistical analyses were carried out using *R* software (version: 3.5.0).

## 3. Results

### 3.1. Demographic Characteristics of Cases and Controls

The demographic characteristics of 818 cases and 935 controls are shown in Table 1. In short, significant differences were not found in age, age at menarche, age at first live birth, and age at menopause. Compared with cancer-free controls, cases were not inclined to higher education levels and household incomes but were prone to a positive family history of breast cancer. Different distributions of body mass index (BMI) and menopausal status were also observed. Of all subjects, 604 (73.84%) cases and 549 (58.78%) controls were postmenopausal, and 465 (57.48%) cases and 449 (48.03%) appeared to be overweight or obese.

### 3.2. Associations between Four SNPs of GC Gene and Breast Cancer Risk

Genotyping assays indicated that the control-group genotype frequencies of four SNPs were in Hardy–Weinberg equilibrium, and call rates of all individuals were above 95% for these four SNPs. The four SNPs genotypes among cases and controls as well as the correlations with risk of breast cancer are presented in Table 2. We carried out linkage disequilibrium analysis of four SNPs and found except for rs16847024, other three SNPs with *r*^2^ > 0.8 ([Supplementary-material supplementary-material-1]). Compared with the AA genotype, logistic regression results showed that AG of rs17467825 was in association with a decreased breast cancer risk (OR = 0.77, 95% CI = 0.62–0.96). For rs2298850, individuals carrying GC genotype could lower the risk of breast cancer compared with the GG genotype (OR = 0.78, 95% CI = 0.63–0.97). Also, the CT genotype of rs3755967 could reduce risk of breast cancer with an OR of 0.77 (95% CI = 0.62–0.96). We also found three SNPs (rs17467825, rs2298850, and rs3755967) with significant association effects on breast cancer under a dominant model after adjusting for selected confounders (rs17467825: OR = 0.80, 95% CI = 0.65–0.99; rs2298850: OR = 0.80, 95% CI = 0.65–0.98; rs3755967: OR = 0.80, 95% CI = 0.65–0.98). However, we failed to find any association of rs16847024 genotypes with breast cancer risk.

### 3.3. Associations between Four SNPs and Breast Cancer Risk by Stratified Analyses

Further subgroup analyses stratified by age, menopausal status, family history, and waist circumference were conducted, and results are shown in Table 3. For rs17467825, rs2298850, and rs3755967, significant reduced risk of these three SNPs with breast cancer was observed among the subgroup of waist circumference <80 cm, the OR (95% CI) = 0.69 (0.51–0.93), OR (95% CI) = 0.66 (0.49–0.89), and OR (95% CI) = 0.67 (0.50–0.91), respectively. Significant heterogeneities were observed in the subgroup of waist circumference (*P*=0.035 for rs17467825, 0.019 for rs2298850, and 0.023 for rs3755967, respectively). Among the subgroups of age, menopause, and family history, there were no statistically significant associations observed.

### 3.4. Interactions of rs17467825, rs2298850, and rs3755967 with Waist Circumference on Breast Cancer Risk

In effort to explore whether or not the identified mutations effects on risk of breast cancer could be modified by waist circumference, we evaluated the interactions of rs17467825, rs2298850, and rs3755967 genotypes and waist circumference. As shown in Table 4, we examined multiplicative interactions between rs2298850 and rs3755967 genotypes and waist circumference on risk of breast cancer (*P* for multiplicative interaction = 0.031 and 0.039, respectively). However, no interaction was detected between rs17467825 and waist circumference on risk of breast cancer (*P* for multiplicative interaction = 0.057). For rs2298850, compared with the waist circumference ≥80 cm with the GG genotype, a significant decreased risk of breast cancer was observed for those with GC or CC genotypes with the waist circumference <80 cm, whereas a nonsignificant increased breast cancer risk was observed for those with the CC genotype with the waist circumference ≥80 cm. The additive interactions between waist circumferences and the SNPs (rs17467825, rs2298850, and rs3755967) were also examined. However, significant additive interactions were not measured between these three SNPs and waist circumferences ([Supplementary-material supplementary-material-1]).

### 3.5. Combined Effects on Breast Cancer Risk

Previous results were mainly focused on the investigation of the association of single SNP with breast cancer risk. Combined analysis was also performed to estimate accumulative effects of these polymorphisms. In Table 5, we identified allele-dosage relation between allele numbers of the protective effect and risk of breast cancer (*P*_trend_=0.043). Compared with subjects carrying “0” protective alleles, individuals with “1–2” alleles of protective effects could possess a nonsignificant 10% (OR = 0.90, 95% CI: 0.37–2.20, *P*=0.816) reduced cancer risk, whereas “3–6” protective alleles could decrease a 19% (OR = 0.81, 95% CI: 0.66–0.99, *P*=0.044) breast cancer risk.

## 4. Discussion

In this work, we estimated the correlations between four genetic variants in GC gene (rs16847024, rs17467825, rs2298850, and rs3755967) and risk of breast cancer in Chinese population and found that heterozygotes of rs17467825, rs2298850, and rs3755967 but not rs16847024 were in significant relation to the breast cancer risk. Also, in the dominant model, three SNPs (rs17467825, rs2298850, and rs3755967) were observed with an approximately 20% decreased breast cancer risk in our study population. Combined analysis also implied that subjects carrying more alleles of the protective effect could decrease the breast carcinogenesis risk.

Epidemiological studies supported that 25(OH)D was related with breast cancer risk [13, 22]. The active vitamin D, 1,25(OH)_2_D, has an effect not only on cell proliferation and apoptosis but also on the estrogen signal pathway [23]. When the circulating 25(OH)D integrated to DBP, the complex, 25(OH)D-DBP, could be internalized in mammary cells by megalin-mediated endocytosis, and the complex internalization had a connection with activation of the vitamin D receptor pathway [24]. In addition, when the complex was internalized, 25(OH)D was released from DBP. In the mammary gland, low concentrations of 25(OH)D might weaken enzyme activity and production of 1,25(OH)_2_D, which regulated cell proliferation and apoptosis [25, 26]. Furthermore, one study has demonstrated that 25(OH)D concentrations played a role in growth inhibition of mammary cells [27].

A breast cancer research in Shanghai showed that about 23% of women involved in study were vitamin D deficiency and 48.4% were vitamin D insufficiency [28]. One study conducted among Chinese pregnant women found that 8 SNPs, including rs17467825, rs2298850, and rs3755967, were in associations with concentrations of 25(OH)D [29]. A case-control study found that women with 25(OH)D < 20 ng/ml and calcium levels < 10.5 mg/dl had higher risk of developing breast cancer [17]. In addition, the biological active vitamin D, 1,25(OH)_2_D, could inhibit breast cancer cell growth by reducing synthesis of estrogen.

Apart from transport function, DBP may also take part in the anticancer process through non-vitamin D-related functions, including roles in chemotaxis and macrophage activation [9]. DBP is thought as a molecule involved in activation macrophages, and it undergoes the deglycosylation process into DBP-macrophage activating factor (DBP-MAF) by glucosidases [30]. DBP-MAF may enhance proapoptotic enzymes activity to induce cell apoptosis via the JNK1/2 and p38 pathway, which may inhibit cancer development [31].

In genome-wide association studies, GC rs2282679 was reported to correlate with serum 25(OH)D levels [32]. Furthermore, GC rs2282679 could reduce the cancer risk [33]. SNPs included in our study (rs17467825, rs2298850, and rs3755967) were in high linkage disequilibrium with rs2282679 (LD = 1.0, 0.9, and 1.0, respectively). Our results may support previous reports that GC polymorphism rs2282679 was associated with cancer risk. The rs17467825 polymorphism is 3′-UTR of the GC gene, rs2298850 and rs3755967 located in the intronic region of the GC gene. Though there are fewer researches on the functional effects of GC polymorphisms, potential functions may not exclude that these genetic variants may take part in regulating gene expression or influencing transcription sites to lead to amino acid modification [34, 35], and this needs further studies to verify. The changes in the structure of GC proteins may alter biological functions involved in carcinogenesis.

A cohort including 28,965 postmenopausal women found that a larger waist circumference was connected to higher breast cancer risk, especially the risk was elevated 13% when the waist circumference increased by 10 cm [36]. We also observed interactive effects between rs2298850, rs3755967 genotypes, and waist circumference on breast cancer risk. These findings suggested that rs2298850 and rs3755967 could modify the susceptibility of breast cancer induced by waist circumference. Gene environment interactions could partly interpret discrepancies in the association between GC gene polymorphisms and breast cancer across different studies. These genetic variants may be worth reevaluating further in other larger studies.

It should be noted that there were several limitations in this study. First, we did not achieve the data about serum 25(OH)D concentration from the study population, so we could not evaluate the influence of 25(OH)D concentration on risk of breast cancer among our study population. Second, only the GC gene was evaluated, and we could not exclude the fact that other genetic variants of vitamin D pathway genes might also have impacts on risk of breast cancer. Finally, this study was carried out among Chinese women. The associations between breast cancer susceptibility and GC genetic polymorphisms in other ethnic populations require further investigation.

## 5. Conclusions

In summary, our study identified some genetic variants of the GC gene associated with risk of breast cancer in Chinese women. Furthermore, we, for the first time, found that there were potential significant interactions between GC gene polymorphisms and waist circumference in susceptibility to breast cancer, thus supporting the insight that genetic variants in conjunction with environmental factors are able to influence individual's cancer risk.

## Figures and Tables

**Table 1:** Characteristics of cases and controls.

Variables	Cases (*n* = 818)	Controls (*n* = 935)	*P* value
	*N* (%)	*N* (%)	
Age (mean ± SD, year)	54.78 ± 11.10	54.27 ± 11.30	0.337
<53	393 (48.04)	458 (48.98)	0.730^b^
≥53	425 (51.96)	477 (51.02)	

Age at menarche (mean ± SD, year)	15.52 ± 2.55	15.48 ± 2.73	0.762^a^
Age at first live birth (mean ± SD, year)	24.60 ± 2.70	24.58 ± 2.48	0.875^a^
Chest circumference (mean ± SD, cm)	90.40 ± 8.69	90.70 ± 7.90	0.451^a^
Age at menopause (mean ± SD, year)	49.18 ± 4.87	49.29 ± 6.04	0.727^a^
Waist circumference (mean ± SD, cm)	87.31 ± 10.98	83.07 ± 9.84	**<0.001** ^**a**^

Menopausal status at baseline			
Premenopausal	214 (26.16)	385 (41.22)	**<0.001** ^**b**^
Postmenopausal	604 (73.84)	549 (58.78)	

Body mass index (mean ± SD, kg/m^2^)	24.93 ± 3.54	24.14 ± 3.16	**<0.001** ^**a**^
<18.5	19 (2.35)	15 (1.60)	**<0.001** ^**b**^
18.5–23.9	325 (40.17)	471 (50.37)	
24.0–27.9	330 (40.79)	339 (36.26)	
≥28.0	135 (16.69)	110 (11.77)	

Education			
Primary school	256 (31.33)	228 (24.44)	**<0.001** ^**b**^
Junior middle school	348 (42.60)	341 (36.55)	
Senior middle school	148 (18.12)	225 (24.12)	
University and above	65 (7.96)	139 (14.90)	

Income (yuan)			
<30000	216 (26.54)	149 (15.94)	**<0.001** ^**b**^
30000–49999	163 (20.02)	121 (12.94)	
50000–100000	265 (32.56)	317 (33.90)	
≥100000	170 (20.88)	348 (37.22)	

Oral contraceptive use			
No	652 (80.10)	760 (82.16)	0.299^b^
Yes	162 (19.90)	165 (17.84)	

Family history of breast cancer			
No	711 (88.76)	873 (94.38)	**<0.001** ^**b**^
Yes	90 (11.24)	52 (5.62)	

^a^Welch's *t*-test was applied for the unequal variances. ^b^Two-sided chi-square test.

**Table 2:** Summary of associations between genetic variants and breast cancer risk.

Genotype	Cases	Controls	Crude OR (95% CI)	Adjusted OR (95% CI)^b^	*P* ^b^
rs16847024 (C > T)^a^					
CC	612	708	1.00	1.00	
CT	175	203	1.00 (0.79–1.26)	0.93 (0.73–1.19)	0.556
TT	13	14	0.99 (0.46–2.16)	0.97 (0.43–2.22)	0.946
CT/TT	188	217	1.00 (0.80–1.25)	0.93 (0.73–1.18)	0.562
Additive model			1.00 (0.81–1.22)	0.94 (0.76–1.17)	0.594

rs17467825 (A > G)					
AA	377	402	1.00	1.00	
AG	325	414	0.83 (0.68–1.02)	**0.77 (0.62–0.96)**	**0.020**
GG	89	93	1.02 (0.74–1.41)	0.95 (0.67–1.35)	0.785
AG/GG	414	507	0.87 (0.72–1.05)	**0.80 (0.65–0.99)**	**0.039**
Additive model			0.95 (0.82–1.09)	0.90 (0.77–1.05)	0.181

rs2298850 (G> C)					
GG	367	393	1.00	1.00	
GC	341	428	0.85 (0.69–1.04)	**0.78 (0.63–0.97)**	**0.025**
CC	89	99	0.96 (0.70–1.33)	0.89 (0.63–1.27)	0.530
GC/CC	430	527	0.87 (0.72–1.05)	**0.80 (0.65–0.98)**	**0.035**
Additive model			0.93 (0.81–1.08)	0.89 (0.76–1.03)	0.123

rs3755967 (C > T)					
CC	372	396	1.00	1.00	
CT	332	420	0.84 (0.68–1.02)	**0.77 (0.62–0.96)**	**0.021**
TT	90	97	0.99 (0.72–1.36)	0.92 (0.65–1.30)	0.627
CT/TT	422	517	0.86 (0.71–1.05)	**0.80 (0.65–0.98)**	**0.035**
Additive model			0.94 (0.81–1.08)	0.89 (0.76–1.04)	0.143

^a^Major allele > minor allele. ^b^Derived from logistic regression with an adjustment for age, menopausal status, BMI, family history, income, waist circumference, and education. OR, odds ratio.

**Table 3:** Stratified analyses of association between 4 SNPs and breast cancer risk.

	Age (years)		Menopausal status		Family history		Waist circumference (cm)	
	<53	≥53	Premenopause	Postmenopause	No	Yes	<80	≥80
rs16847024 (C > T)								
OR (95% CI)^a^	1.00 (0.72–1.38)	0.95 (0.70–1.27)	1.01 (0.69–1.48)	0.92 (0.70–1.20)	0.94 (0.75–1.18)	1.18 (0.50–2.77)	1.04 (0.68–1.58)	0.95 (0.73–1.22)
*P* value^a^	0.983	0.720	0.957	0.517	0.588	0.702	0.863	0.670
*P* for heterogeneity		0.832		0.226		0.613		0.719

rs17467825 (A > G)								
OR (95% CI)^a^	0.84 (0.67–1.06)	0.97 (0.78–1.20)	0.86 (0.65-1-1.14)	0.95 (0.79–1.14)	0.87 (0.74–1.02)	1.23 (0.66–2.28)	**0.69 (0.51–0.93)**	1.01 (0.84–1.21)
*P* value^a^	0.135	0.775	0.289	0.565	0.09	0.512	**0.015**	0.919
*P* for heterogeneity		0.369		0.560		0.289		**0.035**

rs2298850 (G > C)								
OR (95% CI)^a^	0.83 (0.66–1.04)	0.95 (0.77–1.18)	0.86 (0.65–1.13)	0.93 (0.78–1.12)	0.85 (0.73–1.00)	1.23 (0.66–2.27)	**0.66 (0.49–0.89)**	1.01 (0.84–1.21)
*P* value^a^	0.111	0.664	0.271	0.475	0.057	0.512	**0.006**	0.956
*P* for heterogeneity		0.398		0.644		0.255		**0.019**

rs3755967 (C > T)								
OR (95% CI)^a^	0.82 (0.65–1.03)	0.98 (0.79–1.21)	0.84 (0.64–1.11)	0.95 (0.79–1.14)	0.86 (0.73–1.01)	1.22 (0.66–2.26)	**0.67 (0.50–0.91)**	1.01 (0.84–1.21)
*P* value^a^	0.09	0.841	0.226	0.559	0.069	0.532	**0.009**	0.932
*P* for heterogeneity		0.265		0.470		0.284		**0.023**

^a^OR with its 95% CI and *P* value were derived from the additive model (wild-type homozygote vs. heterozygote vs. variant homozygote) using logistic regression adjusted for age, menopausal status, BMI, family history, income, waist circumference, and education.

**Table 4:** Interaction analysis between three SNPs genotypes and waist circumference on breast cancer risk.

Genotypes	Waist circumference (cm)	Cases	Controls	Crude OR (95% CI)	Adjusted OR (95% CI)^a^	*P* ^a^
rs17467825						
AA	≥80	283 (35.8%)	255 (28.1%)	1.00	1.00	
AA	<80	94 (11.9%)	147 (16.2%)	**0.58 (0.43–0.79)**	**0.69 (0.49–0.98)**	**0.037**
AG	≥80	249 (31.5%)	259 (28.5%)	0.86 (0.68–1.10)	0.85 (0.66–1.09)	0.203
AG	<80	76 (9.6%)	155 (17.1%)	**0.44 (0.32–0.61)**	**0.46 (0.31–0.66)**	**<0.001**
GG	≥80	71 (9.0%)	49 (5.4%)	1.31 (0.87–1.95)	1.22 (0.80–1.87)	0.342
GG	<80	18 (2.3%)	44 (4.8%)	**0.37 (0.21–0.65)**	**0.40 (0.21–0.75)**	**0.004**
*P* for multiplicative interaction						0.057

rs2298850						
GG	≥80	272 (34.1%)	251 (27.3%)	1.00	1.00	
GG	<80	95 (11.9%)	142 (15.4%)	0.62 (0.46–0.85)	0.72 (0.51–1.03)	0.069
GC	≥80	264 (33.1%)	270 (29.3%)	0.90 (0.71–1.14)	0.87 (0.67–1.12)	0.280
GC	<80	77 (9.7%)	158 (17.2%)	**0.45 (0.32–0.62)**	**0.46 (0.32–0.67)**	**<0.001**
CC	≥80	71 (8.9%)	51 (5.5%)	1.29 (0.87–1.92)	1.18 (0.77–1.79)	0.443
CC	<80	18 (2.3%)	48 (5.2%)	**0.35 (0.20–0.61)**	**0.37 (0.20–0.70)**	**0.002**
*P* for multiplicative interaction						**0.031**

rs3755967						
CC	≥80	277 (34.9%)	252 (27.6%)	1.00	1.00	
CC	<80	95 (12.0%)	144 (15.8%)	**0.61 (0.44–0.83)**	**0.7 (0.49–0.99)**	**0.045**
CT	≥80	257 (32.4%)	267 (29.2%)	0.87 (0.68–1.11)	0.85 (0.66–1.10)	0.222
CT	<80	75 (9.4%)	153 (16.8%)	**0.44 (0.32–0.62)**	**0.46 (0.32–0.67)**	**<0.001**
TT	≥80	72 (9.1%)	50 (5.5%)	1.31 (0.88–1.95)	1.21 (0.79–1.84)	0.379
TT	<80	18 (2.2%)	47 (5.1%)	**0.35 (0.20–0.62)**	**0.37 (0.20–0.70)**	**0.002**
*P* for multiplicative interaction						**0.039**

^a^Adjusted by age, menopausal status, BMI, family history, income, and education.

**Table 5:** Combined effects on breast cancer risk.

Number of protective alleles^b^	Cases	Controls	OR (95% CI)^*a*^	*P* ^a^
	*N* (%)	*N* (%)		
0	363 (46.30)	390 (43.29)	1.00	
1–2	11 (1.40)	12 (1.33)	0.90 (0.37–2.20)	0.816
3–6	410 (52.30)	499 (55.38)	0.81 (0.66–0.99)	0.044
*P* for trend				0.043

^a^Adjusted by age, menopausal status, BMI, family history, income, waist circumference, and education. ^b^Combined genotypes were according to protective alleles carried (rs17467825-G, rs2298850-C, and rs3755967-T).

## Data Availability

The data used to support the findings of this study are available from the corresponding author upon reasonable request.

## Supplementary Materials

Supplementary Materials (1) Supplementary Figure S1: linkage disequilibrium mapping of four SNPs. (2) Supplementary Table S1: basic information of four SNPs in the GC gene. (3) Supplementary Table S2: additive interaction analysis between rs2298850, rs3755967, and rs17467825 genotypes and waist circumference on breast cancer risk.
