# Supplementary material for: Genetic Variants in Group-Specific Component (GC) Gene Are Associated with Breast Cancer Risk among Chinese Women
Source: Biomed Res Int. 2019 Nov 15;2019:3295781. doi: 10.1155/2019/3295781 (PMC6881756; doi:10.1155/2019/3295781)
Supplement: Supplementary Materials — (1) Supplementary Figure S1: linkage disequilibrium mapping of four SNPs. (2) Supplementary Table S1: basic information of four SNPs in the GC gene. (3) Supplementary Table S2: additive interaction analysis between rs2298850, rs3755967, and rs17467825 genotypes and waist circumference on breast cancer risk. [file 3295781.f1.zip › 3295781.f1/Supplementary Table S1.docx]

**Supplementary Table S1** Basic information of 4 SNPs in GC gene.

| Gene | SNPs | Cases  (WH/H/VH)^a^ | Controls  (WH/H/VH)^a^ | Major/ Minor allele | MAF | MAF  (case/control) | Call rate |
| --- | --- | --- | --- | --- | --- | --- | --- |
| *GC* | rs17467825 | 377/325/89 | 402/414/93 | A/G | 0.3244 | 0.3180/0.3300 | 96.98% |
| *GC* | rs3755967 | 372/332/90 | 396/420/97 | C/T | 0.3298 | 0.3224/0.3363 | 97.38% |
| *GC* | rs2298850 | 367/341/89 | 393/428/99 | G/C | 0.3256 | 0.3253/0.3402 | 97.95% |
| *GC* | rs16847024 | 612/175/13 | 708/203/14 | C/T | 0.1252 | 0.1256/0.1249 | 98.40% |

^a^ Wild homozygous type/Heterozygote/Variant homozygous type.
