# Supplementary material for: Genetic Variants in Group-Specific Component (GC) Gene Are Associated with Breast Cancer Risk among Chinese Women
Source: Biomed Res Int. 2019 Nov 15;2019:3295781. doi: 10.1155/2019/3295781 (PMC6881756; doi:10.1155/2019/3295781)
Supplement: Supplementary Materials — (1) Supplementary Figure S1: linkage disequilibrium mapping of four SNPs. (2) Supplementary Table S1: basic information of four SNPs in the GC gene. (3) Supplementary Table S2: additive interaction analysis between rs2298850, rs3755967, and rs17467825 genotypes and waist circumference on breast cancer risk. [file 3295781.f1.zip › 3295781.f1/Supplementary Table S2.docx]

**SUPPLEMENTARY TABLE S2** Additive interaction analysis between rs2298850, rs3755967 and rs17467825 genotypes and waist circumference on breast cancer risk.

|  | Waist circumferences | | | | |
| --- | --- | --- | --- | --- | --- |
| rs17467825 | <80 | |  | ≥80 cm | |
| Genotypes | No.cases/controls | Adjusted OR (95%CI)^a^ | | No.cases/controls | Adjusted OR (95%CI)^a^ |
| AG+GG | 94/201 | 1.00 |  | 327/318 | 2.05(1.45-2.90) |
| AA | 96/148 | 1.59(1.09-2.32) |  | 283/255 | 2.28(1.61-3.23) |
| Measures of additive interaction | | | | | |
| Relative excess risk due to interaction (RERI) = -0.37 (95% CI: -1.13-0.40) | | | | | |
| Attributable proportion due to interaction (AP) = -0.16 (95% CI: -0.49-0.18) | | | | | |
| Synergy index (S) = 0.78 (95% CI: 0.48-1.25) | | | | |  |
|  | Waist circumferences | | | | |
| rs2298850 | <80cm | |  | ≥80 cm | |
| Genotypes | No.cases/controls | Adjusted OR (95%CI)^a^ | | No.cases/controls | Adjusted OR (95%CI)^a^ |
| GC+CC | 95/206 | 1.00 |  | 335/321 | 2.08(1.48-2.39) |
| GG | 95/142 | 1.63(1.12-2.38) |  | 272/251 | 2.27(1.60-3.21) |
| Measures of additive interaction | |  |  |  |  |
| Relative excess risk due to interaction (RERI) = -0.45 (95% CI: -1.24-0.34) | | | | | |
| Attributable proportion due to interaction (AP) = -0.20 (95% CI: -0.55–0.15) | | | | | |
| Synergy index (S) = 0.74 (95% CI: 0.46-1.18) | | | | |  |
|  | Waist circumferences | | | | |
| rs3755967 | <80cm | |  | ≥80 cm | |
| Genotypes | No.cases/controls | Adjusted OR (95%CI)^a^ | | No.cases/controls | Adjusted OR (95%CI)^a^ |
| CT+TT | 94/205 | 1.00 |  | 332/321 | 2.09(1.48-3.27) |
| CC | 96/144 | 1.64(1.13-2.40) |  | 278/252 | 2.30(1.62-3.27) |
| Measures of additive interaction | | | | | |
| Relative excess risk due to interaction (RERI) = -0.43 (95% CI: -1.22-0.36) | | | | | |
| Attributable proportion due to interaction (AP) = -0.19 (95% CI: -0.52-0.16) | | | | | |
| Synergy index (S) = 0.75 (95% CI: 0.47-1.20) | | | | |  |

^a^ Adjusted by age, menopausal status, BMI, family history, income and education.
