# Supplementary figures and images for: Genetic Variants in Group-Specific Component (GC) Gene Are Associated with Breast Cancer Risk among Chinese Women
Source: Biomed Res Int. 2019 Nov 15;2019:3295781. doi: 10.1155/2019/3295781 (PMC6881756; doi:10.1155/2019/3295781)

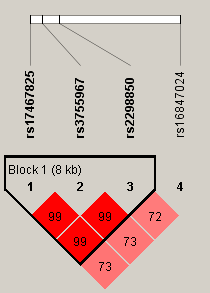

Supplement: Supplementary Materials — (1) Supplementary Figure S1: linkage disequilibrium mapping of four SNPs. (2) Supplementary Table S1: basic information of four SNPs in the GC gene. (3) Supplementary Table S2: additive interaction analysis between rs2298850, rs3755967, and rs17467825 genotypes and waist circumference on breast cancer risk. [file 3295781.f1.zip › 3295781.f1/Supplementary Figure S1.jpg]
